# Are There Lipid Membrane-Domain Subtypes in Neurons with Different Roles in Calcium Signaling?

**DOI:** 10.3390/molecules28237909

**Published:** 2023-12-02

**Authors:** Alejandro K. Samhan-Arias, Joana Poejo, Dorinda Marques-da-Silva, Oscar H. Martínez-Costa, Carlos Gutierrez-Merino

**Affiliations:** 1 Departamento de Bioquímica, Universidad Autónoma de Madrid (UAM), C/Arturo Duperier 4, 28029 Madrid, Spain; oscar.martinez@uam.es; 2 Instituto de Investigaciones Biomédicas ‘Sols-Morreale’ (CSIC-UAM), C/Arturo Duperier 4, 28029 Madrid, Spain; 3 Instituto de Biomarcadores de Patologías Moleculares, Universidad de Extremadura, 06006 Badajoz, Spain; joanapoejo86@gmail.com; 4 LSRE—Laboratory of Separation and Reaction Engineering and LCM—Laboratory of Catalysis and Materials, School of Management and Technology, Polytechnic Institute of Leiria, Morro do Lena-Alto do Vieiro, 2411-901 Leiria, Portugal; dorinda.silva@ipleiria.pt; 5 ALiCE—Associate Laboratory in Chemical Engineering, Faculty of Engineering, University of Porto, Rua Dr. Roberto Frias, 4200-465 Porto, Portugal; 6 School of Technology and Management, Polytechnic Institute of Leiria, Morro do Lena-Alto do Vieiro, 2411-901 Leiria, Portugal

**Keywords:** flotillin, caveolin, ganglioside, lipid rafts, lipid membrane domains, calcium channel, NMDA, PMCA, P2XR, membrane domains, neuron, brain

## Abstract

Lipid membrane nanodomains or lipid rafts are 10–200 nm diameter size cholesterol- and sphingolipid-enriched domains of the plasma membrane, gathering many proteins with different roles. Isolation and characterization of plasma membrane proteins by differential centrifugation and proteomic studies have revealed a remarkable diversity of proteins in these domains. The limited size of the lipid membrane nanodomain challenges the simple possibility that all of them can coexist within the same lipid membrane domain. As caveolin-1, flotillin isoforms and gangliosides are currently used as neuronal lipid membrane nanodomain markers, we first analyzed the structural features of these components forming nanodomains at the plasma membrane since they are relevant for building supramolecular complexes constituted by these molecular signatures. Among the proteins associated with neuronal lipid membrane nanodomains, there are a large number of proteins that play major roles in calcium signaling, such as ionotropic and metabotropic receptors for neurotransmitters, calcium channels, and calcium pumps. This review highlights a large variation between the calcium signaling proteins that have been reported to be associated with isolated caveolin-1 and flotillin-lipid membrane nanodomains. Since these calcium signaling proteins are scattered in different locations of the neuronal plasma membrane, i.e., in presynapses, postsynapses, axonal or dendritic trees, or in the neuronal soma, our analysis suggests that different lipid membrane-domain subtypes should exist in neurons. Furthermore, we conclude that classification of lipid membrane domains by their content in calcium signaling proteins sheds light on the roles of these domains for neuronal activities that are dependent upon the intracellular calcium concentration. Some examples described in this review include the synaptic and metabolic activity, secretion of neurotransmitters and neuromodulators, neuronal excitability (long-term potentiation and long-term depression), axonal and dendritic growth but also neuronal cell survival and death.

## 1. Lipid Membrane Nanodomains Organization in the Neuronal Plasma Membrane

The classical model of the plasma membrane, named the fluid mosaic model, described by Jonathan Singer and Garth Nicolson in 1972, is excessively reductionist for properly accounting for the well-organized plasma membrane domains. Lipid rafts are plasma membrane large areas of 10 and 200 nm diameter in size enriched in cholesterol and sphingolipids [1]. The existence lipid rafts was initially a subject of debate between physical chemists and histologists due to difficulties in visualizing them and their ill-defined molecular composition [1,2]. In the last two decades, a number of new techniques such as single-molecule spectroscopy, super-resolution microscopy, fluorescence recovery after photobleaching, stimulated emission depletion, Förster resonance energy transfer (FRET), total internal reflection fluorescence, and fluorescence correlation spectroscopy techniques allowed to estimate the lower limit of lipid rafts in <20 nm [3,4,5,6]. Plasma membrane domains of 26 ± 13 nm radius have been observed in living cells diffusing as one entity for minutes [7]. Further work using stimulated emission depletion (STED) far-field fluorescence nanoscopy revealed spots sized 70-fold below the diffraction barrier transiently trapped between 10 and 20 ms, in cholesterol-mediated molecular complexes dwelling within <20-nm diameter areas [3]. The diffraction limit of visible light impedes domains smaller than 1 µm to be directly visualized and indeed large micrometer-sized lipid rafts domains are readily observed in artificial membranes [3]. Also, associated proteins can mask the direct observation of lipid rafts in living cells. A tentative attempt to determine analogous domains in living cells has been made based on homo-FRET efficiencies obtained through the rate of fluorescence anisotropy loss and using GFP labeled glycosyl-phosphatidylinositol-anchored proteins which allow an estimation of the upper size limit of lipid rafts at ~5 nm [8,9]. Yethiraj and Weisshaar have suggested that the spatial heterogeneity in cell membranes limits the transferability of the conclusion drawn from artificial membranes to live cells, as integral membrane proteins attached to the cytoskeleton act as obstacles that limit the size of lipid domains [8]. For all these reasons, we introduce the concept of lipid membrane domains in this review, arising from the fact that some membrane proteins form oligomers and clusters in the membranes, which formation is favored by cholesterol and other lipid species.

Regarding the protein components associated with lipid membrane domains, widely named in the bibliography as lipid rafts, a proteomic study identified up to 36 integral membrane proteins associated with lipid membrane domain and flotillin, as a marker of these membrane domains where identified in the human brain [10]. In another study, 175 membrane-associated proteins were identified by proteomics, including L-type calcium channels and the plasma membrane calcium ATPase (PMCA), using caveolin-1 (Cav-1) and flotillin-1 (Flot-1), as biomarkers of lipid membrane domains isolated from brain neonatal mice [11]. Similarly, a proteomic assessment of proteins present in isolated lipid membrane domains of adult mouse brains identified 133 proteins, using Flot-1 as a marker of plasma membrane domains [12]. This study also highlighted the colocalization of this protein with several calcium channel subunits [12]. In cultured hippocampal neurons, sphingolipid-cholesterol-enriched microdomains have been localized flotillin 1, Thy-1 cell surface antigen or CD90, as specific lipid membrane-domain markers, associated with the ganglioside named monosialotetrahexosylganglioside (GM1) [13]. It is worth to mention at this point that although GM1 is not a definite lipid membrane-domain marker, its distribution into lipid membrane domains depends on the concentration. At elevated concentration, GM1 can form its own domains organizing in the plasma membrane in non-lipid membrane-domain areas located predominantly in the L_d_ phase [14]. Very recent discoveries regarding the molecular architecture of lipid membrane nanodomains support their organization in planar tightly packed nanodisks of Cav-1, with a 140Å external diameter size [15]. It is also probable that a similar size supramolecular complex based on flotillin might exist, based on the observed structural conformations of stomatin, prohibitin, flotillin, and the modulator for HflB protease specific for phage lambda cII repressor (HflK/C) domains (SPFH domain) [16]. Also, some studies have reported the isolation of up to 4 types of domains in the plasma membrane at physiological conditions [17]. Given the existence of these nanostructures, a question arises regarding how many of the reported protein molecules in the aforementioned proteomic and non-proteomic studies [10,11,12,13] could fit within a single of these nanostructures on one neuronal lipid membrane domain. The quantity of proteins reported in the neuronal lipid membrane domain contrasts with the number of proteins that could fit within or surrounding a 140Å diameter size nanodisk, if this type of structure stands alone as the main component of neuronal planar-lipid membrane domains in the plasma membrane. Neuronal lipid membrane domains are different from those of the invaginated *caveolae* in a variety of cell types, which require the presence of the protein named cavin and higher-order interactions with other proteins [18,19,20]. Cav-1–cavin interaction seems required to form mature *caveolae*, which have a polygonal shape to induce curvature in non-neuronal cells [21,22,23,24]. Cavin is absent or released when conforming planar-non-invaginated lipid membrane domains [20,25,26,27], like those described in neuronal lipid membrane domains.

In addition to these studies, more efforts are required to ascertain whether Cav-1 nanodisks independently exist in neuronal cells, either as discrete entities supporting non-invaginated areas on the plasma membrane or as components of supramolecular structures analogous to those observed in invaginated *caveolae* [20]. Since supramolecular structures with a similar protein composition to that of *caveolae* do not exist in neurons, the presence of a high number of proteins located in lipid membrane domains raises questions regarding the number of proteins that one Cav-1 nanodisk can hold due to steric hindrance. Methods for lipid membrane-domain isolation based on differential gradient centrifugation cannot discern the existence of lipid membrane nanodomain subtypes. Particularly, cytochemical and histochemical studies combined with physicochemical techniques based on quantitative fluorescence energy transfer (FRET) techniques, as those conducted by the research group led by Prof. Gutierrez-Merino, have provided insights into this matter by identification of proteins in clusters complexing with protein markers of lipid membrane domains (caveolin and flotillin isoforms) at a distance <100 nm in studies performed in neurons and brain tissue using the appropriate secondary fluorescent antibodies against the primary antibody of the selected lipid membrane-domain marker (Box 1) [28,29,30,31,32,33,34,35,36]. As discussed in these articles, this is a particular case of FRET from one donor to multiple acceptors, a situation in which the maximum range of FRET distance is significantly expanded, as analyzed in detail in former studies with purified biological membranes [23,37,38,39]. These research findings might support the existence of clusters that could stand alone as individual entities, such as Cav-1 nanodisks, with a diverse variety of calcium transporter elements. The well-recognized and wide distribution of these transporters in neurons, functioning as partners of lipid membrane-domain markers, strongly suggests the potential existence of multiple lipid membrane-domain subtypes within neurons. A neuronal lipid membrane-domain subtype is defined in this work as a plasma membrane, synaptic or extrasynaptic structure characterized by the presence of a protein biomarker of lipid membrane nanodomain and a specific calcium transport systems. The existence of these subdomains might correlate with the function of calcium gradients associated with cytosolic calcium microcompartments, near the plasma membrane [33,40], and such patterns may arise under certain conditions [41,42,43,44].

In this context, it is intriguing and controversial whether different types of lipid membrane domains might exist within a single cell or across different cell types based on the complex lipid and protein composition of these domains. This issue might be particularly notorious in tissues such as the brain, where recent findings using single-cell sequencing and methods to map the spatial location of gene expression have unraveled the extraordinary cellular diversity existing within this tissue [45]. Strategies for isolating lipid membrane domains, named rafts in these studies, that utilized membrane tension generate large observable membrane domains or lipid rafts, that are converted into small ones when the tension was relieved [17]. This result lends support to the hypothesis that a myriad of not well-described plasma membrane nanodomains might exist.

Box 1 **FRET from one donor to multiple acceptors.**

  Labeling of proteins with donor and acceptor secondary fluorescent antibodies forming a FRET pair is an approach that has been used to identify proteins clustered in lipid raft domains. This is a particular case of FRET from one donor to multiple acceptors because the density of labeling of commercial secondary fluorescent antibodies ranges between 2 and 10 dye molecules per antibody [46,47,48,49], and also because in theory, one primary IgG antibody can bind up to 2 secondary fluorescent IgG antibodies, one in each of the symmetrical domains of the primary antibody. Therefore, one dye molecule of the donor fluorescent antibody can form a FRET pair with 2–10 and 4–20 acceptor dyes bound to the acceptor secondary antibody for 1:1 and 1:2 stoichiometries of the primary/secondary antibody complex, respectively. The major advantage of a high density of labeling of the secondary fluorescent antibody is the amplification of the fluorescence intensity signal for fluorescence microscopy imaging of cells. In addition, it has another collateral advantage for FRET distance calculations, namely, that homotransfer between donors located in one fluorescent secondary antibody and time and space averaging of different orientations of donors and acceptors bound to different IgG molecules which should lead to a distribution close to a random orientation between donor and acceptors.

  The number of acceptor dyes available to a donor dye bound to a fluorescent antibody for FRET will be larger when the target protein units form clusters within lipid membrane-domains. In this case, FRET will extend to acceptor dyes of secondary antibodies bound to the primary antibodies that stain all neighbor protein targets present in the cluster within the area accessible to the IgG complex of primary/secondary antibodies plus the effective FRET distance between the selected donor and acceptor dyes. Each 1:1 complex of primary/secondary IgG antibodies will reach proteins located up to ≈30 nm from the target protein, taking into account the size of IgG molecules and their rotational mobility. Therefore, this implies that donor dyes bound to a primary/secondary IgG/ protein-1 complex can make contacts with acceptor dyes bound to the primary/secondary IgG complex attached to protein-2 separated up to ≈ 60 nm in the same lipid membrane-domain. If there is more than one unit of the target protein-2 stained with the secondary fluorescent antibody labeled with the acceptor dye, the number of acceptors/donor available for FRET will be proportionally increased.

  In addition, the overall rate (k_T_) of FRET can be written for these cases as the sum of the rate of FRET between each one of the possible donor/acceptor pairs that can be formed in the system under study, i.e., k_T_ = Σ k_i_, see for example [50,51]. Therefore, the overall FRET efficiency is the sum of the efficiency of energy transfer between all the possible donor/acceptor pairs that can be formed in the system [50,51,52]. This further increases the effective FRET distance using donor and acceptor secondary fluorescent antibodies. A simple calculation can serve to illustrate this point. For FRET from 1 donor to 10 acceptor molecules located at an equidistant distance, the apparent distance for 50% efficiency of FRET will be ~10 × R_0_ from the target protein labeled with the donor secondary fluorescent antibody, where R_0_ is the value of this distance for a single donor/acceptor pair, which ranges between 5 and 6 for the most frequently used FRET pairs in fluorescence microscopy. Let us remind here that the useful donor/acceptor distance range for a single donor/acceptor pair is approximately up to twice the distance for 50% efficiency of FRET [51,53]. Note that 10 acceptors per donor can be reached in any of the following cases: (i) 1:2 stoichiometry of the primary/secondary antibody complex and an average density of labeling of the acceptor fluorescent antibody of 5 dye molecules per antibody, and only one unit of the target proteins in the lipid membrane-domain; and (ii) 1:1 stoichiometry of the primary/secondary antibody complex and an average density of labeling of the acceptor fluorescent antibody of 5 dye molecules per antibody, with two protein units labeled with the acceptor fluorescent antibody within 60 nm in the same lipid membrane-domain. In summary, the effective FRET distance range extends to 80–200 nm when donor and acceptor dyes are bound to secondary fluorescent IgG antibodies directed against different target proteins present in lipid membrane-domains.

  Thus, FRET using donor and acceptor secondary fluorescent antibodies is a suitable approach to monitor the co-localization of proteins within lipid membrane-domains of 100–200 nm. Also, it follows from this analysis that when there is only one unit of one of the target proteins within each lipid membrane-domain, co-localization of proteins within smaller lipid membrane-domains of 40 or 20 nm can be studied with the use of fluorescent primary antibodies or antibody F_ab_ fragments, respectively, instead of using fluorescent secondary antibodies.

For cells, application of membrane tension resulted in several types of large domains; one class of domains was identified as a lipid raft, defined here as lipid membrane domain. Furthermore, the distribution of protein components of lipid domains [54,55,56,57] in planar non-invaginated regions of the neuronal plasma membrane [20,25,26,27], may be considered a robust evidence for the existence of not-so-transient, underlying structures that support several membrane nanodomains in neurons. This structural arrangement may differ from that observed in other cell types, where membrane invaginated areas forming *caveolae* have been described involved in membrane trafficking, with a transient formation and elimination of the protein content of these domains.

The objective of this review is to provide a comprehensive exploration and integrative analysis of information, suggesting the existence of lipid/protein-domain subtypes within neuronal cells. Several proteins that play major roles in neuronal calcium signaling have been described as components of lipid membrane domains [58], i.e., neurotransmitter receptors [59,60] and calcium transport systems [43,61], and they present a differential subcellular distribution within a single neuron and across different types of neurons, as shown in this review. The distribution pattern serves as a crucial tool for proposing the existence of diverse lipid membrane-domain subtypes in neurons.

## 2. Properties of Caveolin-, Flotillin- or Ganglioside-Containing Lipid Membrane Domains

**Figure 1:**
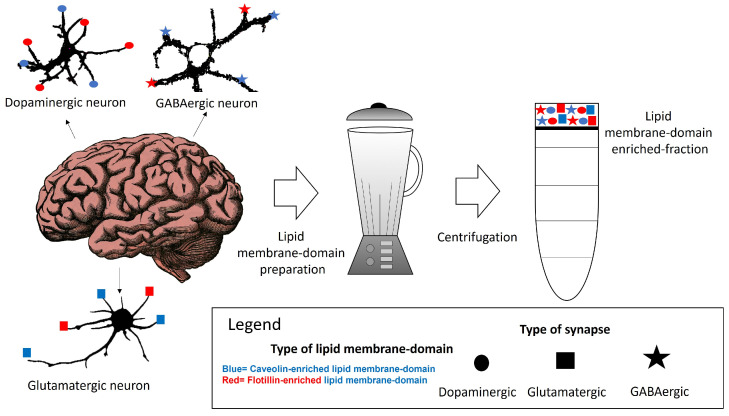
Some of the potential lipid membrane domains that can be isolated from brain tissue are associated with different neuronal types.

Within neuronal lipid membrane domains, at least two classes of protein, named caveolin and flotillin, can scaffold cholesterol and have been used as biomarkers of these domains [62,63,64,65,66,67,68]. The differential spatial distribution of the caveolin-, flotillin- or some specific lipid-enriched domains of the neuronal plasma membrane suggests that various domains co-exist in one neuron. We will call them caveolin- and flotillin-enriched lipid membrane domains. Their differential association with plasma membrane receptors acting through calcium signaling, as well as with calcium channels and transport systems might be useful to classify lipid membrane nanodomains. Other lipids, such as gangliosides have been associated with both in certain contexts but not always [69,70,71]. This supports the idea that their presence might constitute a marker for additional lipid membrane nanodomain subtypes. The characterization and differentiation between these domains have been challenged by the limitations and insufficient resolution of the conventional methods for preparative isolation of lipid domains using a whole brain tissue or cells in culture (Figure 1). This is a major handicap for a proper classification of lipid membrane-domain subtypes. A potential dissection through immunohistochemical and immunocytochemical methods could offer insights of their precise intracellular and intercellular locations. Moreover, this dissection could contribute to a better comprehension of how key plasma membrane components in charge of calcium homeostasis are regulated in lipid membrane domains. The subsequent paragraphs of this review provide a brief account of the actual knowledge of these nanodomains in neurons.

### 2.1. Caveolin-Enriched Lipid Membrane Domains in Neurons

Cav-1 is the major component forming *caveolae* at the plasma membrane [27,72,73,74,75]. Several domains are recognized in the linear sequence of this protein related to its function and its interaction with lipids. Membrane binding, cholesterol recognition, and oligomerization functions have been attributed to the scaffolding domain (SD) of Cav-1 [76,77,78]. As part of the SD, a function for the intramembrane domain (IMD; residues 102−134) has been assigned, forming a unique α-helical hairpin that does not traverse the membrane [79,80,81]. Proteins associated with caveolin are characterized by the presence of an aromatic-rich caveolin binding motif (CBM) with the following compositions (ϕXϕXXXXϕ, ϕXXXXϕXXϕ or ϕXϕXXXXϕXXϕ, where ϕ is an aromatic and X an unspecified amino acid) [82,83,84]. Cav-1 also presents a cholesterol recognition/interaction amino acid consensus (CRAC) domain composed of the amino acid residues VTKYWFYR [85], which allows the interaction of this protein with cholesterol. It must be highlighted at this point that the presence of a CRAC domain in proteins is neither necessary nor sufficient for cholesterol binding [86,87]. In this sense, proteins including CRAC domains can be neutral with respect to cholesterol binding, and proteins lacking CRAC domain can bind cholesterol which is the case of transmembrane protein domains lacking a CRAC, a CARC or a tilted domain, as reviewed by Fantini and Barrantes [88]. For this reason, cholesterol interaction with caveolin might be beneficiated by additional interactions with the protein/membrane microenvironment.

**Figure 2:**
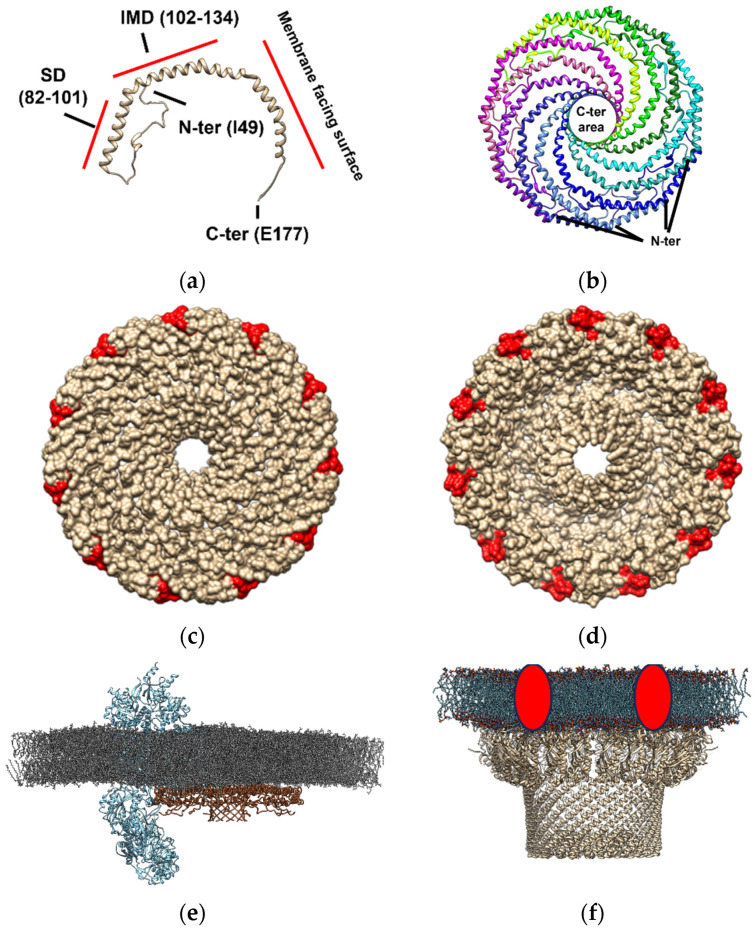
Molecular architecture of caveolin- and flotillin-enriched domains based on Cav-1 forming nanodisks based on the PDB model 7SC0 and as reported in the bibliography [15] and similar proteins to flotillin constituting SPFH domains. Cav-1 constitutes the singular unit for the formation of these structures. The amino and carboxyl end of the protein are labeled as N- and C-end (panel (**a**)). The scaffolding domains (SD, residues 82–101) and the intermembrane domain (IMD, residues 102–134) formed are shown in respect to the Cav-1 region that faces the membrane as shown in this panel. Nanodisks as formed by 11 Cav-1 protomers (individually labeled with different colors) which are tightly packed disks locating in planar membrane-embedded surfaces (panel (**b**)). The location of the C-ends oriented to form a central β-barrel “hub” (~28 Å wide), and N-terminal sides forming an outer “rim” (~23 Å wide) for generation of the nanodisks with a 140 Å diameter in size is shown in this panel. Representation of the membrane-oriented Cav-1 nanodisk surface in respect to cholesterol binding site (labeled in red is shown in panel (**c**). Representation of the cytoplasmic Cav-1 nanodisk surface in respect to cholesterol binding site (labeled in red is shown in panel (**d**)). Location of the cholesterol binding site at the periphery of the nanodisk is compatible with the “lipid belt” proposed model for the interaction of some lipids with ion channels suggesting that cholesterol may be a part of a lipid belt or a “shell” constituting the immediate perimeter of the channel protein with could be mediated by complexation with Cav-1 nanodisks [38,99,100,101]. An artistic representation of a Cav-1 nanodisk (PDB: 7SC0, brown-colored backbone) interacting with voltage-dependent L-type calcium channel subunit α-1S (Cav1.1 subunit, PDB: 5GJW, blue-colored backbone) in a model membrane of dipalmitoyl phosphatidylcholine (colored in grey) is shown in panel (**e**). An artistic representation of the macromolecular structure of a flotillin-enriched domain based on that reported in the literature [102], (using 7VHP PDB model, light-brown-colored backbone) complexing with some proteases that might degrade misfolded/damaged membrane proteins or cytoplasmic proteins (red circles) at the membranes (panel (**f**)). Hydrophobic tails are represented in blue and polar heads in red, as described in bacterial membrane microdomains [102].

Cav-1 (Figure 2, panel a), which is one of the units required for *caveolae* formation, can hetero- or homo-oligomerize in complexes composed of 14–16 monomers (200–400 kDa) [89,90]. Recently, the typical supramolecular structure of this protein has been described by cryo-electron microscopy [15]. Cav-1 overexpression in *E. coli* formed 8S-like complexes and oligomerize, forming heterologous *caveolae* (*h-caveolae*) and sculpting membranes, which are two of the essential functions of mammalian cells *caveolae* [91,92]. Cav-1 can assemble in protomers organized into a tightly packed disc with a planar membrane-embedded surface [15]. Several Cav-1 protomers (11 protomers) can oligomerize to form an 8S complex, a type of complex with a proposed biological role essential for *caveolae* biogenesis since 8S complexes are known to concentrate in endoplasmic reticulum (ER) exit sites [93]. Also, they accumulate at the Golgi, where they lose their diffusional mobility and associate with cholesterol [94,95] and eventually assemble into 70S complexes [93]. The cholesterol-rich membranes containing 70S Cav1 complexes are then transported to the cell surface. The formation of the 8S complex occurs in a cooperative process mediated by its oligomerization domain (OD), which is aided by its SD and signature motif (SM). The crystallography study revealed that the 11 Cav-1 protomers can organize into a disc-shaped complex with a diameter of ~140 Å and a height of ~34 Å to form the 8S complex [15]. The nanodisk contains an outer “rim”, a central β-barrel “hub”, and 11 curved α-helical “spokes” with Cav-1 C-terminal ends oriented towards the hub and N-terminal ends towards the rim (Figure 2, panel b). This study supports that caveolin complexes may stabilize flat membrane surfaces of polyhedral structures rather than imposing continuous membrane curvature [15]. Although this structure is formed in an almost cholesterol-depleted environment, since cholesterol synthesis in *E. coli* is present in only freshly isolated strains [96,97], this study provides evidence of the structural dependence that *caveolae* might have on other proteins but also cholesterol in the membranes [98]. Interestingly, the location of the cholesterol interacting domain on the Cav-1 nanodisk surrounding this structure (Figure 2, panel c and d) is compatible with the “lipid belt” model proposed to mediate the interaction between some lipids and proteins, including ion channels, some of them described as Cav-1 protein partners in lipid membrane domains (Figure 2, panel e). This observation suggests that Cav-1 nanodisks may be a part of a lipid belt or a “shell” constituting the immediate perimeter of the protein channel [38,99,100,101], in those channels where no cholesterol interacting domain has been described, complexing and conforming a lipid-protein membrane domain (Figure 2, panel e).

Regarding caveolin-enriched domains in neurons, certain studies have indicated that neuronal lipid membrane domains associated with caveolin are flat and do not have the invaginated appearance described for *caveolae* [103]. *Caveolae* curvature has been shown to be dependent upon cavin, and its release from lipid membrane domains has been associated with planar non-invaginated surfaces distinct from *caveolae* [20,25,26,27]. The crystallographic studies that provide evidence for the existence of macromolecular structures organized into Cav-1 nanodisks suggest that neuronal lipid membrane domains might at least be constituted by this structure, serving as fundamental units responsible for caveolin-enriched domains present in the plasma membrane of neurons.

Although the best-known endocytic route in cells is dependent upon clathrin and independent upon lipid membrane domains [104,105], alternative endocytic routes involving lipid membrane domains mediated by *caveolae* exist [106,107]. They rely on the protein named dynamin in some cases on Pacsin-2 and are dependent upon cholesterol, as shown by its sensitivity to cholesterol depletion [108,109,110]. They have been involved in the uptake of glycosylphosphatidylinositol (GPI)-anchored proteins (GPI-APs) and opportunistic ligands, including simian virus 40 and cholera toxin (CTx) [111]. Some authors have stated that distinct mechanisms of clathrin-independent endocytosis have unique sphingolipid requirements [112], but in many cases a role has been assigned to caveolin as an initiator of intracellular signaling via protein clustering, the segregation of proteins, and the protein trafficking to and from the membrane-associated with G proteins [113,114]. These processes can generally directly regulate channel permeability for calcium or modulate other components that regulate intracellular calcium concentration through the channel [82,115]. For example, secreted neurotrophins (including brain-derived neurotrophic factor (BDNF) and neurotrophic factors (NT): NT3, NT4, and NT5) can exert prolonged effects on presynaptic transmitter secretion or postsynaptic responses [116]. Neurotrophins binding to their receptors (tyrosine kinase (Trk)-A, Trk-B, Trk-C, etc.) occur in discontinuous regions of neuronal cell membranes associated with membrane lipid membrane domains [117].

Regarding the relevance of caveolin-enriched domains in brain neurons in *in vivo* studies, some of them have shown a correlation between Cav-1–knocking down (Cav-1–KD) and the disruption of Cav-1-enriched membrane domains found in neurodegenerative diseases, such Alzheimer’s disease where an alteration of signaling processes associated with lipid membrane domains has been also described [118]. Caveolin has also been implicated in synaptic vesicle exocytosis impairment ascribed to changes in synaptic vesicle dynamics driven by Cav-1 palmitoylation using a Cav-1- knock-out animals (Cav-1-KO) [119]. Oppositely, an increase in caveolin expression was found to improve and preserve motor and cognitive function after brain trauma using animal models [120]. These experiments support that Cav-1 levels might enhance cellular survival and growth. Also, some researchers support its role as a candidate for its level modulation to repair the injured and neurodegenerative brain [121,122]. The opposite effect has been observed in some animal models of Huntington’s disease, where a loss or reduction of Cav-1 expression rescues the phenotype in neurons and significantly delays the onset of motor decline and development of neurons. Therefore, aberrant interaction between Huntingtin and Cav-1 leading to altered cholesterol homeostasis in these diseases has been suggested [123].

### 2.2. Histological and Cytological Distribution of Caveolin-Enriched Lipid Membrane Domains in Neurons and Their Function in Calcium Signaling

Cav-1 has been identified as a component of lipid membrane domains localized within cell bodies and dendrites of primary culture of cerebellar granule neurons and Purkinje cells [33,119,124,125], soma and postsynapses of the anterior cingulate cortex neurons in tissue [126,127], cell body and *puncta* localized to areas of cellular outgrowth and synapses and dendritic spines of primary culture of hippocampal neurons [128,129,130].

A study has shown that Cav-1 partially colocalize with the N-methyl D-aspartate receptor subtype 2B (NR2B) subunit of the N-methyl-D-aspartic acid receptor (NMDAR), which is highly enriched in dendritic shafts and spines of rat cortical neurons at postsynaptic terminals [131,132]. NMDARs are glutamate-gated ion channels that mediate excitatory neurotransmission in the central nervous system (CNS) [133]. The presence of NMDARs at presynapses or postsynapses has a different function. In the case of presynapses, NMDA receptors have a function in neurotransmission and plasticity [134], and postsynaptic receptors are needed for spike-timing-dependent long-term depression (LTD) induction [135]. A study of Cav-1 overexpression in neurons showed that Cav-1 mediated expression of NMDAR subtypes promoting pathways dependent upon the membrane cholesterol associated with primary neuron arborization events [121]. Two regions on NR2B subunits (W^635^AFFAVIF^642^, and, F^1042^SFKSDRY^1049^) have been potentially suggested to interact with caveolin-binding motifs [84,132]. A disruption of the interaction between Cav-1 and NR2B has anti-nociceptive effects at the anterior cingulate cortex [126], which correlate with the observed effect of pain agonists promote a shift of the NR2B subunits of NMDA receptor subunit to non-lipid membrane-domain areas [132]. Also, an increased amount of caveolin promotes an enhanced surface level of NR2B in this brain area [126], which leads to an increase in cytosolic calcium concentration and activation of extracellular-signal-regulated kinase/cAMP response element (ERK/CREB) signaling pathways [136]. Thus, decreased caveolin expression in cells disrupts NMDAR signaling events, and reintroducing Cav-1 rescues proper NMDAR signaling. Since NR2B contains the binding site for glutamate [137], this suggests that caveolin is required for the signal transduction pathway activated by glutamate release from the presynaptic terminals [132]. It has been suggested that the regulation of the NR2B subunit by Cav-1 might be attributed to the modulation of proto-oncogene tyrosine-protein kinase (Src) activity since Cav-1 was observed to be essential for NMDA-mediated phosphorylation of Src and ERK1/2 activation [132], which is required for NMDA-mediated signaling (i.e.,: NMDA preconditioning stimuli) [121,132,138].

Src family tyrosine kinases (SFKs) serve as central regulators for the modulation of NMDAR signaling in normal and ischemic conditions and the induction of long-term potentiation (LTP) [139,140,141]. This modulation accounts for SFK-mediated tyrosine phosphorylation of NR2B, a subunit found highly phosphorylated in postsynaptic terminals [140,141]. Head and collaborators proposed that Cav-1, via its ability to scaffold key signaling components, mediates in the NMDAR localization to neuronal membrane domains, NMDAR/Src tyrosine kinase family/ERK signaling, and protection of neurons from ischemic injury and cell death [132]. Cav-1 promotes NR2B surface levels and has been shown to contribute to the modulation of chronic neuropathic pain in the anterior cingulate cortex [126].Cellular stress events (i.e., superoxide anion radical, osmotic stress, and UV exposure) can increase SFK-mediated phosphorylation of caveolin [142]. In addition, some studies early reported the existence of a negative regulatory feedback loop in non-neuronal cells in which Y14 phosphorylated Cav-1, would bind and activates C-terminal Src kinase (Csk) and subsequently phosphorylates and inactivates Src [143,144,145,146]. In neurons, the regulatory role of Cav-1 phosphorylation/dephosphorylation by Src/Csk has been shown to mediate axonal outgrowth of motor neurons in Xenopus neuromuscular development [147]. Regarding the regulation of the system by oxidative stress, it should be noted that indeed it can activate both Src-kinases and their negative regulator Csk and induces phosphorylation of Cav-1 as a targeting protein for Csk [145]. These results suggest that caveolin could mediate in events mediated by NMDAR, such as those associated with neuronal plasticity and injury that might be associated with oxidative stress [132,148], by regulation of its level of phosphorylation.

Presynaptic NMDA receptors play pivotal roles in excitatory neurotransmission, contributing to synaptic plasticity and facilitating presynaptic neurotransmitter release, functions that are crucial for synaptic maturation and plasticity during formative periods of brain development [134,149,150]. It has been reported that presynaptic NMDA receptors might modulate superoxide anion production by NADPH oxidases (NOXs) [151]. In turn, NMDA receptors may be modulated by superoxide anion by a similar mechanism in postsynapses [142], and locations where NR2B subunits have been found at presynapses as the *cerebellum* [152] and neocortex [153]. In this case, modulation by superoxide anion might be associated with superoxide anion producing enzymes of very specific sources, also clustering within lipid membrane domains [32]. Flavoproteins, such as the enzyme cytochrome *b*_5_ reductase (C*b*_5_R), have been established to form complexes within plasma membrane lipid membrane domains of cerebellar granule neurons, as those described by our laboratory [28]. C*b*_5_R is one of the major sources of superoxide anion in the plasma membrane lipid membrane domains of cerebellar granule neurons [29,31]. This protein holds the potential to facilitate certain superoxide anion-dependent adjustments of the NMDA receptor at the presynaptic terminals. The existence of these proteins associated with caveolin [33], might constitute an alternative form of caveolin-enriched lipid membrane-domain subtype in respect to those previously commented.

Both C*b*_5_R and neuronal nitric oxide synthases (nNOS), as alternative redox flavoproteins located within the neuronal plasma membrane lipid membrane domains, have been proposed to form complexes associated with caveolin-enriched domains [32,33]. These complexes have been postulated to function as redox nanotransducers, in charge of controlling calcium transporters such as the L-type calcium channels and NMDA receptors. These microchip-like structure have been proposed to tightly orchestrate coupling between calcium and nitric oxide signaling in presynapses of glutamatergic cerebellar granule neurons (CGNs) [32]. The co-localization of these components agrees with the suggested effect of glutamate on the activation of NMDA receptors in neuronal terminals containing nNOS, leading to nitric oxide (NO^•^) formation and amplifying neurotransmitter release, a mechanism early hypothesized by Snyder and Dawson [154]. These specialized domains can promote a localized and transient increase in calcium concentration up to 1 µM within a nearby microcompartments of 100 nm with low calcium buffering capacity [32]. nNOS is inactive at low calcium concentrations, but it active when calcium concentration is high enough to afford a significant saturation of calmodulin (EC_50_ ≈ 0.2–0.4 μM). The mechanism by which nNOS is regulated by caveolin remains unknown. The modulation of nNOS activity by Cav-1 seems to be distinct from the one observed to regulate endothelial NOS [155].

In hippocampal and cortical neuron cultures, α-amino-3-hydroxy-5-methyl-4-isoxazolepropionic acid receptor (AMPAR) has been associated with caveolin-enriched lipid membrane domains [156]. The AMPAR present in lipid membrane domains is regulated by the activity of NMDAR and NO^•^-mediated pathways [129,156]. This regulation might be potentially interconnected with redox nanotransducers described above in adjacent domains observed in cerebellar granule neurons, particularly in presynaptic membranes [32]. NO^•^ has a mimicking similar effect to that of NMDA, leading to the recruitment of AMPARs to the surface since lipid membrane domains are required for receptor insertion into the membrane [156]. Cholesterol depletion leads to instability of surface AMPAR, a gradual loss of synapses (both inhibitory and excitatory), and loss of dendritic spines [129].

Metabotropic glutamate receptors (mGluRs) are responsible for so-called slow synaptic transmission associated with the effects of peptide neurotransmitters and non-peptide neuromodulators [157,158]. Metabotropic receptors are G-protein-associated receptors enriched at excitatory synapses [159,160]. There are eight subtypes of mGluRs classified in presynapses as group I mGluRs (formed by mGluR1/mGluR5 subtypes) selectively activated by 3,5-dihydroxyphenylglycine and coupled to inositol phospholipid hydrolysis, group II mGluRs (formed by GluR2/mGluR3 subtypes) and group III mGluRs (formed by mGluR4/mGluR6/mGluR7/mGluR8 subtypes) [161,162,163]. Association of metabotropic mGluR with caveolin has been shown for group I and II [159,164,165], which might have a wide different location depending on the receptor type [166]. These group I of metabotropic glutamate receptors are modulated by Cav-1 [128] through the caveolin binding motif of the mGlu1 receptor (FVTLIFVLY-ϕXXXXϕXXϕ). Cav-1 interacts with mGluR1 through a motif contained within the last segment of the first transmembrane (TM) domain and the first intracellular loop of the receptor [159]. A second putative Cav-1 binding motif contained within i3 and the first segment of TM6 is also present in mGluR1/5 [159]. Localization of mGluR1/5 in lipid-protein membrane domains is promoted by Cav-1, which controls the rate of constitutive mGluR1 internalization and, therefore, regulates the expression of the receptor at the cell surface [128,159]. Indeed, the control of constitutive mGluR internalization rate and the surface level of mGlu1 has been shown to be dependent upon caveolar/lipid membrane-domain-dependent endocytosis associated with Cav-1 [159]. In addition, activation of other mGluR induced through complex to estrogen receptor subunits has been associated with different caveolin isoforms, including Cav-3 expression, in different brain areas: *striatum*, the estrogen receptor (ER) alpha (Erα)/Cav-1/mGLUR5/Gq GTPases (Gq) complex and the ERα or ERβ/Cav-3/mGLUR3/Gi/o proteins complex; *hippocampus*, ERα/Cav-1/mGLUR1a/Gq complex and the and the ERα or ERβ/Cav3/mGLUR2/Gi/o complex; the *arcuate nucleus*, ERα/Cav-1/mGLUR1a/Gq; astrocytes (*hypothalamus*) ERα/Cav-1/mGLUR1a/Gq; dorsal root ganglion neurons, ERα/Cav3/mGLUR2/Gi/o [167].

A G-protein-dependent intracellular calcium release by activation of phospholipase C (PLC), inositol-3-phosphate (IP3) pathway, and the transient receptor potential canonical channel (TRPC) are components associated with the group I of metabotropic receptors. These proteins are all present in lipid membrane domains [168,169,170,171]. Using dihydroxyphenylglycerol, an agonist of the group I mGluR, an increase in the mGluR1α clustering level to phosphorylated caveolin was found [172]. Other studies have shown that, the interaction between Cav-1 and group I mGluRs regulates mGluR-dependent phosphorylation/activation of MAPKs [159]. Lipid membrane-domain disruption with methyl-β-cyclodextrin induced a block in the agonist-dependent mGluR1α internalization, being the implication of caveolin suggested in synaptic plasticity in the *cerebellum* [173].

L-type calcium channels are known to regulate synaptic activity, contributing to the initiation of endosome recycling, which regulates the abundance of synaptic molecules such as AMPA-type glutamate receptors in neuronal dendrites [174]. This function might support the existence of L-type calcium channels associated with caveolin-enriched domain as a lipid membrane nanodomain subtype located at postsynaptic membranes [174]. Some subunits of the L-type calcium channel, such as A2δ-2 subunits, colocalize with proteins binding to gangliosides in alternative lipid membrane-domain structures to those described associated with caveolin [175]. Although non-invaginated caveolar structures have been suggested to exist in neurons, internalization of neurotrophins activated tyrosine kinases receptors (TrkA) [176] and TrkB [118], at growth cones might be dependent upon caveolin-associated endocytosis [177,178]. L-type calcium channels are also very sensitive to oxidative stress, as reported by the NMDA receptor, but in this case, by direct effect since these complexes present an allosteric thiol-containing “redox switch” that controls the activity of the L-type calcium channel [179].

Regulation of N-type calcium channel by Cav-1 has been observed in caveolin-enriched lipid membrane domains of neuroblastoma NG108-15 cell lines [180]. Downregulation of Cav-1 production in these cells induced a 79% reduction in the N-type current density without significant changes in the channel’s activation and inactivation time course. The regulation of the channel by membrane cholesterol associated with caveolin was observed to be responsive to this effect rather than induced by direct modulation by caveolin [180]. A similar modulatory effect was observed for R-type voltage calcium channels and neurokinin receptors using kidney cell lines, where cholesterol was responsible for its modulation since intracellular diffusion of Cav-1 scaffolding peptide or overexpression of Cav-1 unaffected the channel function [180].

Localization of the PMCA has also been found at caveolin-enriched lipid membrane domains [33]. The cerebellar synaptosome isoform 4 of the PMCA was specifically localized in this domain with respect to other isoforms locating at non-lipid membrane domains [181]. Some studies show the stimulation of PMCA by acidic phospholipids such as phosphatidylserine [182]. This lipid is normally located at the inner leaflet of plasma membranes and enriched in caveolin-enriched domains in non-neuronal cells [183]. Phosphatidylserine externalization is typical of cell death processes associated with apoptosis [184], and this event might modulate PMCA activity and the interaction this lipid [185].

Purinergic receptors (P2X) have been associated with Cav-1-enriched lipid membrane domains [186,187,188]. Cooperatively, CaMKIIα and Cav-1 drive ATP-induced membrane delivery of the P2X3 receptor as reported in dorsal root ganglion neurons [187]. The NH_2_-terminus of the P2X3 receptor was identified to interact with caveolin through the ‘T^12^KSVVVKSWTI^22^’ motif and the extended motif ‘F^6^FTYETTKSVVVKSWTI^22^’ was engaged to CaMKIIα binding [187]. P2X3 receptors are associated with calcium influxes, which further activate the calcium/calmodulin-dependent protein kinase IIa (CaMKIIa), and are primarily expressed in primary sensory neurons located in dorsal root ganglion (DRG) responsible for pain [189,190]. Upon receptor phosphorylation, an increase in P2X3 interaction with Cav-1 has been observed, providing a mechanism for P2X3 receptor sensitization in pain development [187]. It is particularly noteworthy that immunoreactivity of P2X3 in the plasma membrane was not decreased by the cholesterol depletion with methyl-β-cyclodextrin and cholesterol sequestering had no effect on P2X3- or P2X2/3-mediated inward currents [191]. This result support that the P2X3 receptor may be diffusely distributed in lipid membrane domains and in non-lipid membrane domains in primary sensory neurons [191].

### 2.3. Flotillin and Neuronal Lipid Membrane Domains

Domains formed by flotillin in the plasma membrane differ from those in which Cav-1 is present. Furthermore, they are dynamic and bud into the cell [192]. The main protein components of these domains are the flotillin isoforms, Flot-1 and Flot-2, which share 50% sequence identity [193]. They are in charge of membrane curvature induction in non-neuronal cells, the formation of plasma-membrane invaginations morphologically similar to *caveolae*, and the accumulation of intracellular vesicles [192]. Early studies suggested flotillin proteins organization into stable tetramers in membrane microdomains [194]. Some studies suggested the possible role of flotillin as a new marker of *caveolae* [194], and subsequent studies have shown that flotillin and caveolin do not always co-localize [56]. Nevertheless, it cannot be discarded that a certain amount of flotillin could be enriched at *caveolae* [195]. An estimation of the size dimension of flotillin-enriched lipid membrane domains by immunolabelling suggests the formation of patches ranging 40–200 nm in neurons [196]. These studies correlate with a description of flotillin protein complexes as part of a family of proteins named SPFH (stomatin, prohibitin, flotillin, and HflK/C) forming an operon with NfeD proteins [197]. The ancient origin of SPFH-domain proteins and the Nodulation efficiency protein D (NfeD) protein and the stomatin operon partner protein (STOPP) can be traced back to the ancient living cells that diverged and evolved to *Archaea* and *Bacteria* to constitute the main binding region of apolar polyisoprenoids as well as cholesterol, contributing to lipid membrane-domain formation [197].

SPFH are proteins enriched in the plasma membranes and also in other subcellular membranes, of prokaryotic and eukaryotic cells [111,198]. Electron microscopy studies have shown a wide distribution of Flot-1 in cells localizing at the cytoplasmic side of the plasma membrane, the cytoplasmic side of primary and secondary lysosome membrane, lipofuscin, multivesicular bodies, Golgi saccules, the cytoplasmic leaflet of the vesicles associated with Golgi apparatus and the lumen side of ER of neuronal cells of rat brain [196]. They have an SPFH domain in common in their structure formed by an N-terminal hydrophobic region that associates proteins to the membrane [111,198]. Flotillin isoforms contain a conserved domain C-terminal to the SPFH domain, called the ‘flotillin domain’, although is not present in the other SPFH domain-containing proteins [193]. SPFHs can form high ordered structures complexes organized as circular structures comprising homo- or hetero-oligomers [102,199,200]. Several structural membrane microdomain organizations by SPFH family proteins have been proposed [102] (Figure 2, panel f). In flotillin structure, two domains with unclear functions have been shown to be present. The first SPFH domain contains sites for acylation [201,202]. In contrast, the C-terminal domain mediates the oligomerization and contains Ala-Glu repeats and phosphorable Tyr residues [203,204,205], which are important for flotillin function.

In brain, anatomical and physiological studies have shown that Flot-1 enhances the formation of glutamatergic synapses but not GABAergic synapses, and it has been suggested that this protein might have a role in neurodevelopmental disorders and axon regeneration and growth [206]. Flotillin is recognized as essential for growth cone elongation and regeneration in retinal ganglion cells and mouse hippocampal neurons [207,208]. Notably, when flotillin isoforms are downregulated, and the signaling pathways that govern actin dynamics are disrupted, axon formation fails to occur [209].

Some studies have demonstrated that flotillin directly regulates the formation of cadherin complexes [210,211]. Flotillin-enriched domains have been observed to be required for the dynamic association, stabilization of cadherins at cell–cell junctions [212], transducing extracellular signals into intracellular signaling events, and modulating alterations in the cytoskeleton in response to various external stimuli [213], signal transduction of Trk receptors, and participates in cellular trafficking pathways [214]. However, the molecular mechanism of action of this protein in these processes is not well understood [215].

It is known that Flot-1 acylation determines this protein traffic from the endoplasmic reticulum toward the plasma membrane [210]. Palmitoylated Flot-1 efflux from the endoplasmic reticulum also mediates Cav-1 traffic to the plasma membrane, avoiding the endoplasmic reticulum stress by inhibiting the synthesis of Cav-1 [210]. Once Flot-1 reaches the plasma membrane, it hetero-oligomerizes with Flot-2 and undergoes depalmitoylation/repalmitoylation, which evokes prolonged insulin-like growth factor-1 (IGF-1) signaling [210]. Recently, a role of Flot-1 in mediating the membrane expression and cellular responses of the transient receptor potential vanilloid type 2 (TRPV2) has been described in primary neuronal culture of dorsal root ganglion [216]. This suggests a crosstalk between TRPV2 and lipid membrane-domain components may influence the cellular morphology and play critical roles in nociception and pain [216]. Also, flotillin depalmitoylation has been linked to receptor cycling between the plasma membrane and endosomes alone or with Flot-2 [210].

Although palmitoylation/palmitoylation of flotillins regulate this protein location into lipid membrane domains, the regulatory role of palmitoylation is not exclusive for this protein. Cav-1 can be palmitoylated on multiple cysteine residues although palmitoylation is not necessary for localization of caveolin to *caveolae* [217]. Palmitoylated Cav-1 has been involved in signaling molecules assembly in plasma membrane *caveolae* and in intracellular cholesterol transport [218]. Also, cav-1 palmiltoylation for example, can regulate synaptic vesicle dynamics events [119], which are processes associated with SNARE machinery [219] linked with different plasma membrane domains [220]. Some of the proteins constituting the SNARE complexes might eventually be associated with lipid membrane domains [221,222]. Therefore, this process should not be directly associated with lipid membrane domains [223].

Glebov and collaborators have suggested flotillin participation in a third endocytosis pathway different from those described for clathrin and caveolin [224]. Flot-1 can colocalize in endosomes with the fluid-phase marker dextran, the glycosylphosphatidylinositol-anchored CD59 (GPI-AP CD59), and CTx and is required for a dynamin-independent endocytic pathway that mediates receptor-independent fluid-phase endocytosis and these markers [224]. This supports that gangliosides colocalization might be used to track endocytosis processes, as also suggested for caveolin-enriched lipid membrane domains. In neurons, flotillin was initially discovered in caveolin-independent cholesterol- and glycosphingolipid-enriched membrane microdomains expressed during axon regeneration [212].

### 2.4. Histological Cytological Distribution of Flotillin-Enriched Lipid Membrane Domains in Neurons and Function Calcium Signaling

Flotillin isoforms have been widely used as a lipid membrane-domain biomarker. Flotillin isoforms have been observed to colocalize with calcium channel α1 subunit CaV2.1, which are subunits of P/Q type calcium channels located presynaptic areas of the brain [175,225], GPI-enriched areas [226] and small uniform *puncta* of pre and postsynapse of hippocampal neurons [206,227], soma and postsynapses of rat cerebral cortex [127]. Flotillin-enriched lipid membrane domains are abundant in the axonal plasma membrane and are found in less amount in somatodendritic membranes [228]. This correlated with electrophysiological results using whole-cell patch clamp, showing that Flot-1 increases in the frequency of miniature excitatory postsynaptic currents but not miniature inhibitory postsynaptic currents. In contrast, amplitude and decay kinetics of either type of synaptic current were unaffected, linking these domains with calcium homeostasis [206].

One-third of the NMDAR clusters with flotillin in cultured hippocampal neurons [227]. In hippocampal neurons, both NR2A and NR2B subunits of NMDARs interact with Flot-1 [227]. Flot-1 has been associated with the NR1 subunit preferentially at synaptic areas rather than non-synaptic NR1-enriched areas of hippocampal neurons [206]. It has been suggested that NMDAR interaction with flotillin is involved in recruiting NMDARs into lipid membrane domains to initiate second messenger signaling cascades linked with receptor depletion for neuronal protection during NMDAR-induced excitotoxicity [229]. Indeed, some lipoprotein receptor involved in cholesterol traffic from astrocytes to neurons, such as low-density lipoprotein receptor-related protein 1 (LRP1) [230,231,232], has been suggested to influence the composition of postsynaptic protein complexes through NMDA-induced degradation of the postsynaptic density protein 95 (PSD-95) [233], which might link this process with cholesterol homeostasis and regulation of lipid membrane domains enriched on PSD-95. NMDARs can associate with scaffold protein PSD-95 and form signaling complexes that differ in composition depending on whether they are found in the postsynaptic density or the presynaptic lipid membrane domains. Recently, enhancement in the formation of glutamatergic synapses but not gamma-aminobutyric acid-dependent (GABAergic) synapses has been observed by modulation of Flot-1 level, which suggests further exploration of Flot-1 effect in neurodevelopmental disorders [206]. The authors have postulated that flotillin might have a role in the endocytic internalization of the NMDA receptors after high neuronal stimulation, thereby implicating a subtype of flotillin-enriched domain in the modulation of this process [227]. Flot-1 acylation determines this protein traffic from the endoplasmic reticulum toward the plasma membrane and supports the idea that these domains might be involved with the trafficking of these receptors toward the membrane [168].

Flot-1 and Flot-2 are associated with Ras-binding family of small GTPase 11A (Rab11A) and sorting nexin 4 (SNX4) binding proteins that participates in the recycling and co-transportation of PSD-95, N-cadherin, the glutamate receptors GluA1 and GluN1 to be delivered to the postsynaptic membrane in spines of hippocampal neurons [234]. The mechanism of action remains to be determined [234].

The Cav 2.1 subunit (also known as α (1A) subunit) is a component of the P- and Q-type calcium channels [235], which have different locations and properties than the L-type calcium channels associated with caveolin domains. The α2δ-2 subunit of P- and Q-type calcium channels [236,237,238], partitions with Cav2.1 subunit into flotillin-enriched lipid domains isolated from the *cerebellum* [175].

PMCA has also been found in isolated flotillin-enriched lipid membrane domains from dissociated cortical and hippocampal primary neurons in culture, and its activity has been affected by cholesterol depletion [181,239]. The PMCA activity in these domains has been described to be higher than the PMCA activity excluded from these microdomains [240]. The activity decreased when cholesterol was depleted from these domains [240].

### 2.5. Gangliosides as a Lipid Membrane-Domain Biomarkers for Some Caveolin- and Flotillin-Enriched Lipid Membrane Domains

The presence of gangliosides has been observed in both caveolin- and flotillin-enriched lipid membrane domains [241,242,243], although they are not specifically localized at the plasma membrane and their properties are not exclusively dependent on their polar head group [244]. This type of lipid is strongly abundant in the brain, i.e., in cerebellar granule neurons, they are 5% of total amphipathic lipids [245]. The resulting ganglioside-driven membrane organization are reliant on its production pattern, which is tightly regulated [244]. Not all gangliosides colocalize at the same type of plasma membrane domains [246]. Some authors have concluded that proteins binding to plasma membrane gangliosides can be divided into host plasma membrane proteins and extracellular proteins [247]. Some gangliosides such as GM1 are known to be particularly enriched in the outer leaflet of neuronal lipid membrane domains and exhibit a nearly exclusive presence within these domains compared to non-lipid membrane domains regions. The lipid membrane domain/non-lipid membrane domain ratio values range from 10 to 1000 [248]. Recent molecular dynamics simulation data have shown that three different subpopulations of gangliosides such as GM1 can be characterized in the same lipid membrane domain [14,249], distributed into the central, peripheric and edge areas, which defines their mobility from less to high [247]. Gangliosides at the edge adopt the typical chalice or butterfly-like (open wings) dimeric conformation [250], although conformational possibilities might be further extended by the biochemical diversity of gangliosides. Ganglioside concentration in the same lipid membrane domain creates a large negative electrostatic surface potential, which is one of the essential properties of lipid membrane domains for protein, toxin, or pathogenic agents easily binding due to the electropositive potential [247].

Two types of gangliosides binding domains (GBD) have been described in proteins present in lipid membrane domains:

- Type 1 GBD, or GBD-1, comprises any membrane protein ganglioside-binding domain able to form a stoichiometric (1:1, mol:mol) complex with a single ganglioside molecule [247]. GBD-1 is generally present at the flexible juxta membrane region interacting with transmembrane glycoproteins [113]. The serotonin 5-HT1A receptor, the tumor stem cell marker CD133 are candidates the EGF and PDGF receptors and ion transporters [247]. These membrane proteins are expected to reside at the edge of a lipid raft.
- Type 2 GBD, or GBD-2 are represented by protein dimeric structures resembling a flower chalice or the open wings of a butterfly [250,251]. The typical protein insertion processes have been associated with these domains in which proteins with a hairpin loop interact with the ganglioside, leading to a conformational change that implicates a deep interaction with the ganglioside [251]. This type of ganglioside-dependent insertion process accounts at the edge of a lipid raft or at the periphery since they need to have sufficient conformational flexibility to accommodate the loop [251]. Chalice-shaped ganglioside dimers are required for HIV fusion with host cell membranes [247,252] and the formation of oligomeric calcium permeable amyloid pores [247,253].

In this organization, it is unclear which proteins present in flotillin and caveolin-enriched domains, and more specifically in the brain, might contain GBDs. Cav-1 and Flot 1 have been shown to colocalize with 5-hydroxytryptamine receptor (5-HT1A) [254], and CD133 colocalize with Cav-1-enriched lipid membrane domains [255], which present GBD-1. Caveolin, but no flotillin [256], has been associated with HIV infection and latency [257,258], and this might correlate with the presence of HIV proteins associated with GBD-2 domain. Increased GM1 concentrations have been found in cerebrospinal fluid ganglioside, indicating neuronal involvement in all stages of HIV-1 infection [259].

### 2.6. Histological Cytological Distribution of Gangliosides-Enriched Lipid Membrane Domains in Neurons and Function Calcium Signaling

Regarding calcium transport systems, gangliosides are well-known modulators of calcium homeostasis [260]. PMCA2 and 3 are known to be regulated by endogenous ganglioside content, such as the asialoGM1 that promotes a decrease in pump activity [261,262]. This correlates with the identification of PMCA location in caveolin-enriched lipid membrane domains in cerebellar granule neurons by Marques-da-Silva and Gutiérrez-Merino [33]. The highest PMCA activity is present in the lipid membrane domains enriched in cholesterol and gangliosides [263], which correlates with a report showing that neuraminidase treatment and D-threo-1-phenyl-2-decanoylamino-3-morpholino-1-propanol (d-PDMP), a used inhibitor of glycosphingolipid biosynthesis, induce a decrease in PMCA activity [261]. However, the mechanism of PMCA inhibition by GM1 is still under discussion. Some researchers have suggested that GM1 affects the PMCA interaction via calmodulin modulation of calcium pump affinity and the *V*_max_ [262]. This contrasts with the suggestion of modulation based on the interaction with the calmodulin-binding domain stimulating the phosphatase activity of PMCA by stabilizing E(2) conformer [264,265]. Total lipid membrane domains associated PMCA activity is higher than the PMCA activity excluded from lipid membrane-microdomains [240]. Depletion of cellular cholesterol dramatically inhibited the activity of the lipid membrane-domain-associated PMCA with no effect on the activity of the non-lipid membrane-domain pool [240]. This modulatory function of gangliosides contrasts with that inducing activation of L-type calcium channels, as shown in N18 neuroblastoma cells by the same gangliosides [266].

An almost complete colocalization of NMDARs with the lipid membrane-domain marker ganglioside GM1 has been found in postsynaptic densities close to GM1 [267]. GM1 has been shown to reduce the neurotoxicity of NMDAR, which suggests that receptors located at this location might differentially response to glutamate in this location. However, GM1 does not suppress the function of the NMDAR channel directly [268,269,270]. This protection might be associated with endocytic internalization of the NMDA receptors associated with flotillin-enriched lipid membrane domains, as indicate above [227].

By electron microscopy, a subpopulation of synaptic membrane fractions has been found to be enriched in GM1, and 46 percent of the labeled vesicles are also labeled the GluR2 subunit of the AMPAR [271]. SFKs has been associated with gangliosides and caveolin-enriched lipid membrane domains [272]. They are important since they also mediate the phosphorylation of the AMPARs [273], and they can mediate GluA2-binding protein exchange through endocytosis of GluA2-containing synaptic AMPARs [60]. This might constitute an additional subtype of lipid membrane domains enriched in gangliosides and implicated in endocytic processes or the same associated with Src and NMDA receptors at excitatory synapses. Location studies suggest that AMPAR within PSD are segregated from NMDA receptor clusters [274,275]. In addition, a study has shown that GM1-bound to GluR2-containing AMPARs are functionally segregated from the AMPAR-trafficking complexes (ATCs) containing Thorase, n-ethylmaleimide-sensitive factor attachment protein gamma (γ-SNAP), N-ethylmaleimide sensitive fusion protein (NSF), and nicalin bind selectively to trisialoganglioside gt1b (GT1b) [276], which could define alternative AMPAR domains at the plasma membrane.

GM1 modulation of calcium channels was first described in neurons using N18 neuroblastoma cells [266,277,278] and primary neurons [279,280]. Studies with N18 cells showed that GM1 blocked the intracellular calcium increase sensitive to dihydropyridine blockers at a concentration of 5 mM [266], proposing GM1 function as a constitutive inhibitor of L-type calcium channels [260]. GM1 functions as neuritogenic molecules in neuronal differentiation phases [278]. Upregulation of this lipid has been found in the plasma and nuclear membranes during axonogenesis [278]. In the presence of neuraminidase (N’ase), an enzyme that increases the cell surface content of GM1, a prolific outgrowth of neurites has been found in Neuro-2a and NG108-15 cells [278]. This effect can be blocked by the cholera toxin B, a biochemical tool extensively used for labeling lipid membrane domains using fluorescent conjugates, which potentiated the effect of N’ase in NG108-15 cells [278].

Although cholera toxin binding to ganglioside GM1 supports that this regulation is mediated by lowering free GM1 concentration in the plasma membrane, it remains to be known whether cholera toxin can be sequestered the GM1 localizing in lipid membrane domains, which might modulate the L-type calcium channels associated with these domains. Neurite outgrowth correlated with the influx of extracellular calcium, which correlates with the reported modulation of calcium channels by gangliosides [260].

Using synaptosomes, the N-type calcium channels has also been found to be activated by GM1 ganglioside, followed by the P-type, and very weakly influencing other channels in cerebrocortical synapses [281]. Based on previous indications showing gangliosides with association with caveolin- and flotillin-enriched lipid membrane domains, it is not clear if calcium transporter elements modulated by this lipid might constitute a population implicated in the endocytic process or just be simply subjected to endocytosis.

## 3. The Summary of the Distribution Map

**Figure 3:**
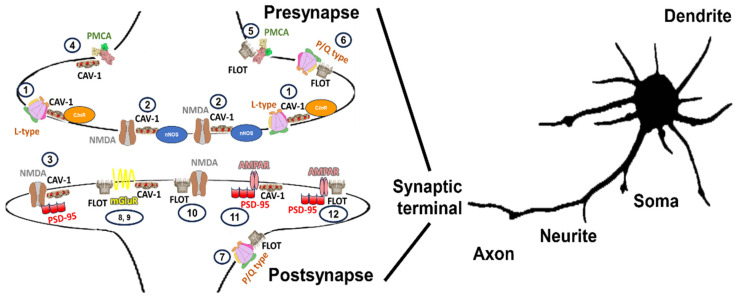
Illustration of a variety of caveolin- and flotillin-enriched lipid membrane domains location complexing with calcium transporter elements (NMDAR, L-P/Q calcium channels, some metabotropic receptors, and PMCA) in synaptic terminals described to exist in glutamatergic neuronal cells. Calcium transporter elements have been differentially described to be present in many neuronal locations, including somas, neurites, axons, dendrites, spines, and synaptic terminals. In synaptic terminals a variability of subunits may yield specific calcium transporters for that location (i.e.,: presynaptic and postsynaptic NMDAR might be differentiated by the type of subunits that configure them in hippocampal neurons [282]) that might differ in configuration from those distributed in other neuronal locations and vary in respect to the neuronal cell type [135]. In this figure, we are focusing on calcium-transporting elements associated with caveolin- and flotillin-enriched lipid membrane domains, that should be added to those elements that are not located in lipid membrane-domain areas (not shown in this figure) and omitted in synaptic terminals that comprise areas of 0.5 to 2 μm size [283]. Lipid membrane domains associated with gangliosides are suggested to be involved in endocytic processes in some membranes and have been omitted from this figure for the sake of clarity. NMDA (1) and L-type calcium channels (2) located in caveolin-enriched domains might function as redox nanotransducers in charge of the control of these calcium transporters working as a microchip-like structure for a tighter functional coupling between calcium, nitric oxide and superoxide anion signaling in presynapses [32,154,155] and postsynapses (3) also sensitive to superoxide anion [132,142], in glutamatergic CGNs. Also, in associated caveolin-enriched domains at presynapses, we can allocate PMCA (4), which are susceptible of inhibition by GM1 contained in these subdomains [33,261,262]. PMCA has also been described to be present in flotillin-enriched lipid membrane domains (5), which are very sensitive to cholesterol content. The activity in these domains is higher than the one not present in lipid membrane domains [240]. The differential response to endogenous cholesterol and gangliosides seems to support that caveolin and flotillin-enriches domains constitute different lipid membrane nanodomain subtypes in presynaptic terminals. We can also find P/Q-type calcium channels at presynaptic terminals (6) associated with flotillin-enriched domains and GPI-enriched areas [175,225,226]. This type of cluster can also be found in postsynaptic terminals (7) [206], and the physiological behavior has been characterized by the presence of Flot-1 and has been related to and increases in the frequency of miniature excitatory postsynaptic currents. Several subunits of metabotropic receptors have been described to colocalize in caveolin- and flotillin-enriched domains (8) and (9). Subunit interaction with caveolin has been better described than for flotillin. Several motifs of mGlu subunits have been described to interact with cav-1 [128,159], which controls the rate of receptor internalization and location at the surface [128,159]. A function of recruitment of NMDARs into lipid membrane domains at postsynapses to initiate second messenger signaling cascades linked with receptor depletion for neuronal protection in NMDAR-induced excitotoxicity has been suggested for NMDAR located at flotillin-enriched domains (10) [212]. As previously indicated, NMDARs can associate with scaffold protein PSD-95 and form signaling complexes that differ in their composition. Some subunits of the AMPAR have also been located in caveolin- (11) and flotillin-enriched domains (12) at post synaptic terminals associated with PSD-95. NO^•^ has a similar effect mimicking that of NMDA, recruiting AMPARs to lipid membrane-domain surface which suggest a counterplay with lipid membrane domains associated with postsynaptic domains (3) or presynaptic (1) and (2) domains since NO^•^ can reach this location by diffusion from presynaptic sources.

A wide range of possible complexes enriched in lipid membrane nanodomain subtypes in the same or different glutamatergic neurons has been described. The organization of NMDAR, L-P/Q calcium channels, some metabotropic receptors, and PMCA located in the synapses of glutamatergic neurons are shown in Figure 3.

A summary of the components implicated in calcium signaling in neurons and their association and function with each lipid membrane-domain subtype can be found in the Table 1.

## 4. Conclusions

Future work should further elucidate the relationship between caveolin- and flotillin-enriched domains and the proteins and lipid partners present in each type of platform that, as shown in this review, may form different lipid membrane-domain subtypes. This includes the effect of cholesterol in calcium signaling and the potential modulation of elements in charge, including calcium channels, that might differentially interact with this lipid in neurons, concerning the same population of protein that might present in non- lipid membrane-domain areas. The cumulative experimental evidence analyzed in this review suggest that lipid membrane-domain subtypes are likely to exist in neurons, largely based on the well-known location and distribution of calcium transporter elements differentially interacting with caveolin- and flotillin-enriched domains.

There is a need for a better characterization of the molecular components of different lipid membrane-domain subtypes in different types of neurons, and of the role of protein-protein and protein-lipid interactions in the functional modulation of the components of these domains. One of the open questions to be answered is associated with the role of cholesterol and its effects, induced by direct interaction with proteins or by changes in the physical-chemical properties of the membranes. Cholesterol enantiomers are potential tools that might help to answer this question since they have identical physical properties to cholesterol but opposite three-dimensional configurations compared to cholesterol [300]. An additional question that needs to be addressed in the future concerns the presence of proteins such as cavin that are present in *caveolae* of non-neuronal cells and seem to be required for the plasma membrane curvature. Neuronal plasma membranes are non-invaginated, suggesting that cavin is not present in these structures, but it should not be discarded the presence of other Cav-1 homologous partners at the neuronal lipid membrane domains that have been determined to be present in non-neuronal cells such as caveolin-2 (Cav-2). Cav-2 is a protein that has also been located at neuronal plasma membrane lipid membrane domains. Indeed, antibodies against this protein have been shown to be helpful in inhibiting some of the protein activities associated with plasma membrane lipid membrane nanodomains of synaptosomes, such as C*b*_5_R activity [29,31]. Although an antagonist role has been described for Cav-2 with respect to Cav-1 due to the ability of Cav-2 to bind cholesterol [301], it cannot be discarded the presence of Cav-2 in the same domains or its role as a major component of some lipid membrane nanodomain subtype. Besides calcium transport channels, the majority of the proteins associated with lipid membrane domains are lipid-anchored proteins [302,303]. Cholesterol might also modulate the dynamics of bulk phases in membranes, altering membrane proteins’ folding and stability, and impacting energetics for protein oligomerization [304]. The hypothetical role of recently discovered molecular architectures enriched in caveolin-forming nanodisks [15], in buffering, distributing, or controlling cholesterol availability for neuronal plasma membrane proteins deserves to be studied in future studies.

## Figures and Tables

**Table 1:** Calcium signaling components and distribution map in lipid raft-domain subtypes.

Type	Subunit	Neuronal Type	Associated with Raft Component	Main Distribution in Brain and Subcellular Location	Function
L-type	Cav1.2	Primary culture of cerebellar granule neurons and Purkinje cells [30,279]	Cav-1 and GM1 [30], GM1 [279]		Neuronal calcium transients in cell bodies and dendrites, regulation of enzyme activity, regulation of transcription [125]
P/Q-type	Cav2.1	Cerebellar Purkinje neurons (tissue [175]; primary culture [284]; brain synaptosomal fraction [225])	Flot-1 [175], GM1 [225,284]	Hippocampus [285], dorsal root ganglion neurons [286], presynaptic areas [225,286]	Neurotransmitter release, dendritic calcium transients [125]
L/P/Q/N-type	α2δ-2, α2δ-3 [226]	Hippocampal neurons (raft isolation and microscopy) [226]	Flot-1 [226]	GPI-enriched areas [226]	
NMDA	NR1	Primary cultures of hippocampal neurons [206]; ganglion cells in rat retina (tissue) [287,288]; ventral part of lamina III and in laminae III and IV [289]	Flot-1 [206]; GM1 [287,288,289]	Small uniform puncta throughout the neuron, pre and postsynapse [206,289]; ganglion cell dendrites [287], extrasynaptic plasma membrane [288]	Signaling complexes in the postsynaptic density [290], glutamatergic signaling, synaptic plasticity, excitotoxicity, and memory [132], neurite outgrowth and axonal growth cone motility [291,292]
	NR2B	Anterior cingulate cortex neurons in tissue and cultured (microscopy and immunoprecipitation) [126]; neurons from normal rat cerebral cortex (raft isolation, microscopy and immunoprecipitation) [127]; primary culture of cortical neurons (microscopy and raft isolation) [132]; ganglion cells in rat retina (tissue) [287,288]	Cav-1 [126,127], Flot-1 [127]; GM1 [287,288]	Soma and postsynapses [126,127]; ganglion cell dendrites extrasynapses peri-synapses [287,288]	
	NR2A [227]	Cultured hippocampal neurons (microscopy and raft isolation) [227]	Flot-1 and -2 [227]	Small uniform puncta throughout the neuron [227]	
AMPAR	GluA2 [130]	Primary culture of hippocampal neurons (microscopy, immunoprecipitation and raft preparation) [130]	Cav-1 [130],	Cell body and as puncta localized to areas of cellular outgrowth [130]	Postsynaptic currents mediated by the AMPA subtype of glutamate receptors in LTP [293]; long-term potentiation (LTP) induced GluA1 surface exposure [294]
	GluA1 [156,234]	Primary culture of hippocampal neurons (microscopy and raft isolation) [156,234]	Flot-1 and -2 [234], Cav-1 [129], GM1 [156]	Postsynapses [156], synapses and dendritic Spines [129]	
	GluR2/3 [129]	Primary culture of hippocampal neurons (microscopy) [129], synaptosomes [271]; ganglion cells in rat retina (tissue) [287]	Cav-1 [129], GM1 [271,287]	Synapses and dendritic spines [129]; dendrites and somata [287]	
	GluR4	Ganglion cells in rat retina (tissue) [287]	GM1 [287]	Dendrites and somata [287]	
mGluR	mGluR1/5	Primary hippocampal neurons (microscopy and immunoprecipitation) [128]	Cav-1 [128]	Soma and dendrites [128]; postsynaptic density late in development [295]	Synapse formation and plasticity [159]
	mGluR1a	Hippocampus, *arcuate nucleus*, *hypothalamus* [167]	Cav-1 [167]		Caveolin proteins act to functionally isolate distinct estrogen receptors and mGluRs, leading to activation of specific second messenger signaling cascades [167]
	mGluR1α	Synaptosomes from pig *cerebellum*	Cav-1 and Flot [173,248]		By application of MβCD, interaction of phosphorylated caveolin with the receptor decreased, and finally, internalization of the receptor was blocked [173]
Pumps	PMCA isoform 4	Synaptosomes from pig *cerebellum* (Brij96 extracts) [181]	ganglioside GM1 [181]		Discrete functional positions on the synaptic nerve terminals [181]
Purinergic receptors	P2X3	Rat brain, cerebellar granule neurons in culture (microscopy, immunoprecipitation and raft preparation), dorsal root ganglion neurons in culture	Flot-2, Cav-1	P2X3 subunit is expressed in cell bodies as well as in peripheral and central terminals of sensory neurons in dorsal root ganglia (DRG) [296,297]	Well-defined role in pain perception [298,299]. Cav-1 is required for basal and ligand-induced membrane delivery of the P2X3 receptor [187]

Note: The reason for no data regarding some of the calcium components and the main distribution in brain and subcellular location is the description of these calcium components in experiments performed *in vitro* in culture. Although some of these cultures were prepared from tissue, we thought this should be differentiated from histochemical studies reporting calcium transported elements in rafts directly visualized on tissue slices or directly prepared or isolated from those tissues.

## Abbreviations

5-hydroxytryptamine receptor (5-HT1A); NSF attachment protein (γ-SNAP); α-amino-3-hydroxy-5-methyl-4-isoxazolepropionic acid receptor (AMPAR); AMPAR-trafficking complexes (ATCs); brain-derived neurotrophic factor (BDNF); calcium/calmodulin-dependent protein kinase IIa (CaMKIIa); caveolin-1 (Cav-1); Cav-1–knocking down (Cav-1–KD); caveolin-2 (Cav-2); cytochrome *b*_5_ reductase (C*b*_5_R); cerebellar granule neurons (CGN); caveolin binding motif (CMB); central nervous system (CNS); Cholesterol Recognition/Interaction Amino Acid Consensus (CRAC); C-terminal Src kinase (CsK); cholera toxin (CTx); dorsal root ganglion (DRG); estrogen receptor (ER); fluorescence energy transfer (FRET); ganglioside binding domains (GBD); monosialotetrahexosylganglioside (GM1); glycosylphosphatidylinositol (GPI); trisialoganglioside gt1b (GT1b); insulin-like growth factor-1 (IGF-1); intermembrane domain (IMD); inositol-3-phosphate (IP3); long-term depression (LTD); low-density lipoprotein receptor-related protein 1 (LRP1); metabotropic glutamate receptors (mGluRs); neuraminidase (N’ase); modulation efficiency protein D (NfeD); N-methyl-D-aspartate receptor (NMDAr); neuronal nitric oxide synthase (nNOS); NADPH oxidases (NOXs); N-ethylmaleimide sensitive fusion protein (NSF); NMDAr subtype 2B subunit (NR2B); oligomerization domain (OD); purinergic P2X receptor (P2XR); protein data bank (PBD); phospholipase C (PLC); plasma membrane calcium ATPase (PMCA); postsynaptic density protein 95 (PSD-95); scaffolding domain (SD); Src tyrosine kinase family (SFK); signature motif (SM); stomatin, prohibitin, flotillin, and HflK/C domains (SPFH); stomatin operon partner protein (STOPP); transmembrane (TM); tyrosine kinase receptors (Trk); transient receptor potential canonical channel (TRPC); transient receptor potential vanilloid type 2 (TRPV2).
